# Revision Rotator Cuff Repair With Versus Without an Arthroscopically Inserted Onlay Bioinductive Implant in Workers’ Compensation Patients

**DOI:** 10.1177/23259671231175883

**Published:** 2023-06-06

**Authors:** Ryan S. Ting, Yao Chen Loh, Ron Rosenthal, Kaitlin Zhong, Hilal S.A. Al-Housni, Mina Shenouda, Lisa Hackett, Patrick H. Lam, George A.C. Murrell

**Affiliations:** * Orthopaedic Research Institute, Saint George Hospital Campus, University of New South Wales, Sydney, Australia.; *Investigation performed at the Orthopaedic Research Institute, St George Hospital Campus, University of New South Wales, Sydney, Australia*

**Keywords:** biological augmentation, bioinductive implant, biological patch, patch, collagen implant, rotator cuff

## Abstract

**Background:**

The addition of onlay biological grafts to augment difficult rotator cuff repairs has shown encouraging results in a case series.

**Purpose/Hypothesis:**

The purpose of this study was to determine whether the addition of an onlay bioinductive implant would improve repair integrity, shear wave elastographic appearance of the repaired tendon and patch, and patient-rated and/or surgeon-measured shoulder function when used in workers' compensation patients undergoing revision arthroscopic rotator cuff repair. We hypothesized that the addition of the bioinductive implant would enhance repair integrity and clinical outcomes compared with standard repair.

**Study Design:**

Cohort study; Level of evidence, 3.

**Methods:**

A post hoc matched-cohort study was conducted on prospectively recruited workers’ compensation patients who received a bioinductive implant for revision rotator cuff repair (n = 19). The control group was selected from consecutive workers’ compensation revision rotator cuff repair patients before the introduction of bioinductive implants. Then, they were matched for age and tear size (n = 32). Kaplan-Meier curves were generated to compare the primary outcome of repair integrity between groups. The secondary outcomes were to evaluate the elastographic appearance of the tendon and patch in the bioinductive implant group and to compare patient-rated and surgeon-measured shoulder function between groups preoperatively and at 1 week, 6 weeks, 3 months, and 6 months postoperatively.

**Results:**

No major complications associated with the bioinductive implants were identified. Six months after the revision rotator cuff repair, the retear rate in the bioinductive implant group was 16% (3/19), compared with 19% (6/32) in the age- and tear size-matched control group (*P* = .458). At the final follow-up, the retear rate in the bioinductive implant group was 47% (9/19) at a mean of 14 months compared with 38% (12/32) at a mean of 29 months in the control group (*P* = .489). The shear wave elastographic stiffness of repaired tendons augmented with the bioinductive implant remained unchanged at 6 m/s from 1 week to 6 months postoperatively, which is lower than the stiffness of 10 m/s in healthy tendons. There were no significant differences in patient-rated or surgeon-measured outcomes between groups 6 months postoperatively.

**Conclusion:**

There were no differences in repair integrity or clinical outcomes between workers’ compensation patients who underwent revision arthroscopic rotator cuff repair with an onlay bioinductive implant compared to those who underwent standard revision rotator cuff repair.

Retear of a surgically repaired rotator cuff tendon is a common problem with an even greater risk in patients undergoing revision rotator cuff repairs.^14^ Shamsudin et al^18^ compared 50 revision rotator cuff repair patients with 310 primary rotator cuff repair patients and found that the likelihood of retear was >1.75 times greater at 6 months and >2.5 times greater at 2 years in patients who underwent revision surgery than in patients who underwent primary repair.

We hypothesized that the increased retear rate may be due to biological factors that lead to decreased healing in patients undergoing revision rotator cuff repair. One potential source of additional healing is the addition of a bioinductive material. Bioinductive implants offer little to no structural support. Rather, bioinductive implants in canine and ovine models have been shown to serve as scaffolds for fibroblast ingrowth and neotendon formation. The subsequent infiltration of native cells may then be followed by the reconstitution of the material properties of the tendon.^1,22^

Initial attempts at adding a bioinductive implant were compromised by xenograft reactions.^23^ More recent studies, however, have reported low rates of xenograft reactions, high rates of graft healing, and improved clinical outcomes in patients with partial-thickness rotator cuff tears treated with onlay bioinductive implants, without rotator cuff repair.^2,17^ However, partial-thickness rotator cuff tears treated with standard repair do extremely well, with retear rates reported as low as 5%. Large full-thickness tears and revision rotator cuff repairs are more problematic.^12^

Thon et al.^19^ however, reported encouraging results for the use of bioinductive implants in 23 patients who underwent arthroscopic repairs of large-massive full-thickness rotator cuff tears, augmented with bioinductive collagen implants after capsular release. They found that 96% (22/23) of the repairs had healed by 2 years, with evidence of new tendon formation on ultrasound and magnetic resonance imaging (MRI). However, this study was a case series with no control group.

We hypothesized that the addition of an arthroscopically inserted onlay bioinductive implant would enhance rotator cuff tendon healing, resulting in better repair integrity at 6 months postoperatively and beyond. The aims of the present study, therefore, were (1) to determine whether the use of an onlay bioinductive implant improved repair integrity, (2) to evaluate the appearance of the repaired tendon and bioinductive implant using **shear wave elastography (SWE) **, and (3) to compare patient-rated and surgeon-measured shoulder function in consecutive workers’ compensation patients who underwent revision arthroscopic rotator cuff repair with an onlay bioinductive implant compared with a control group of workers’ compensation patients matched for age and tear size who did not receive bioinductive implants.

## Methods

This was a post hoc matched-cohort study. Consecutive workers’ compensation patients who underwent revision arthroscopic rotator cuff repair using the Regeneten Bioinductive Implant (Smith & Nephew) formed the intervention group. The use of onlay bioinductive implants in our community was only funded by workers’ compensation for revision rotator cuff repair. The control group was selected from consecutive revision rotator cuff repair patients also funded by workers’ compensation and matched for age and tear size. Ethics approval was granted for this study. Patients provided informed consent.

### Inclusion and Exclusion Criteria

Patients who required revision arthroscopic rotator cuff repairs were eligible for enrollment. Patients were included if they were (1) >18 years old, (2) had approved workers’ compensation claims, and (3) had an ultrasound-confirmed full-thickness retear of a previously repaired supraspinatus tendon that was arthroscopically reparable without the need for interposition grafts, superior capsular reconstruction, or arthroplasty.

Patients who had (1) irreparable rotator cuff tears, (2) partially reparable rotator cuff tears, (3) rotator cuff tears repaired with a synthetic patch, (4) isolated subscapularis tears, (5) rotator cuff repair associated with calcific tendinitis, (6) associated fractures, or (7) concurrent stabilization procedures were excluded from this study. Patients who did not attend the 6-month postoperative ultrasound assessment of repair integrity were also excluded.

### Matching Protocol

Tear size and age are the strongest independent predictors of retear.^11^ Therefore, before further data analysis, the control group was matched based on age by excluding patients whose ages lay outside the age range of those in the bioinductive implant group. The mean tear size area and age were compared between the bioinductive implant group and the control group using the Mann-Whitney *U* test to ensure that there were no statistically significant differences in tear size area or age between the 2 groups.

### Surgical Technique

#### Rotator Cuff Repair

All rotator cuff repairs were performed arthroscopically by the senior author (G.M.). Patients were positioned in the upright beach-chair position and received an interscalene block and sedation. An arthroscope was inserted into the glenohumeral joint through a posterior portal. Debridement of the tendon and footprint was performed using an arthroscopic shaver. A knotless inverted mattress technique was performed using a suture passer (OPUS SmartStitch; Smith & Nephew) and secured with knotless suture anchors (Opus Magnum 2; Smith & Nephew).^7,20^

#### Bioinductive Implant

**Figure 1.**
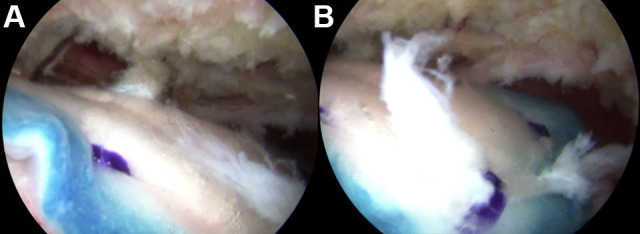
Arthroscopic images of the (A) central portion and (B) lateral edge of a fixed bioinductive implant overlying a repaired supraspinatus tendon, visualized from the posterior portal.

The Regeneten bioinductive implant is a highly porous type I bovine collagen scaffold that is designed to facilitate the migration of fibroblasts and promote collagen formation and remodeling.^22^ The implants used in the present study were 20 × 25 mm in area and 2 mm in thickness. Patients in the bioinductive implant group subsequently received a prerolled Regeneten bioinductive implant that was shuttled through a delivery system centered around a guidewire that was tapped into the humerus 5 to 7 mm lateral to the footprint of the supraspinatus tendon. The delivery system was then deployed, which unfurled the implant. Subsequently, 5 to 6 tendon anchors were inserted to fix the lateral, anterior, and posterior borders of the rectangular implant to the underlying tendon, with the lateral edge of the implant aligned adjacent to the lateral edge of the tendon. The delivery system was then removed, and 2 to 3 anchors were then deployed to fix the medial edge of the rectangular implant to the underlying tendon (Figure 1).

### Postoperative Care

Patients used a sling with an abduction pillow (UltraSling; DJO) for 6 weeks. From day 1 until 6 weeks postoperatively, pendular reach, elbow flexion and extension, grip, and scapular exercises were recommended. From day 8 until 6 weeks postoperatively, shoulder external-internal rotation as well as flexion and extension exercises were recommended. Active shoulder movements and isometric exercises were recommended from week 6 postoperatively. Overhead activity and lifting >5 kg were allowed after 3 months. Patients returned for follow-up visits at 1 week, 12 weeks, and 6 months postoperatively.

### Data Collection

#### Intraoperative

Operative times were measured using a digital clock (minutes), starting at the skin incision and ending after the skin closure. The tear size area (cm^2^) was the product of anteroposterior by mediolateral tear size (cm), which was measured intraoperatively. The diameter of an arthroscopic shaver was used as a reference, as previously described.^20,21,24^

#### Postoperative

All patients completed a standardized 12-item functional questionnaire that was modified from the L’Insalata Shoulder Rating Questionnaire^13^ preoperatively and at 1 week, 6 weeks, 3 months, and 6 months postoperatively (AppendixFigure A1). Patients were asked to self-rate the frequency of pain with activity, sleep, and extreme pain (never, monthly, weekly, daily, always); level of pain at rest, with overhead activity, and during sleep; difficulty with reaching behind the back and reaching overhead, respectively (none, mild, moderate, severe, very severe); stiffness (not at all, a little, moderately, quite, very); and an overall rating of their shoulders (very bad, bad, poor, fair, good). In addition, they provided their level of work (none, light activity, moderate activity, strenuous labor) and sport (none, hobby sport, club sport, national sport). Return to work was defined as a level of work greater than “none” (ie, light, moderate, or strenuous).

Passive shoulder range of motion (ROM) in forward flexion, abduction, external rotation, and internal rotation, respectively, and shoulder strength in abduction, adduction, internal rotation, external rotation, and lift-off from behind the back were measured using a handheld dynamometer (HFG 110; Transducer Techniques) preoperatively and at 6 weeks, 3 months, and 6 months postoperatively.^6,11,16,20^

### Ultrasound Assessment

All assessments of rotator cuff repair integrity and SWE stiffness of the supraspinatus tendon were performed on an ultrasound system with virtual touch imaging quantification elastography (Siemens Acuson S3000 HELIX Evolution ultrasound system with Virtual Touch IQ; Siemens Medical Solutions) with a linear 9L4 MHz transducer, per previously validated protocols.^4,9,21^ A single sonographer (L.H.) with 25 years of experience in shoulder ultrasound performed all assessments. The integrity of the supraspinatus tendon was evaluated using grayscale (B-mode) ultrasound. Patients were seated with their shoulders at 35° of extension, elbows at 90° of flexion, forearms in supination, and dorsum of their hands resting on their ipsilateral thigh. The ultrasound probe was then placed in longitudinal view to diagnose rotator cuff retear before revision surgery as well as postoperatively at 1 week, 6 weeks, 3 months, 6 months, and every visit thereafter. After 6 months, patients were either invited to return for a follow-up or were re-evaluated for various reasons, including problems with their contralateral shoulder. These patients were offered an ultrasound assessment, and the data from these assessments were added to the study.

To assess the SWE stiffness of the supraspinatus tendon, the Virtual Touch IQ creates a color-coded elastogram in which the color bar displays the minimum and maximum range of the shear wave velocity (m/s). On the color display, blue represents low velocity values (0.05 m/s), whereas red represents high velocity values (10 m/s). On this ultrasound system, the 9L4 transducer has a shear wave velocity range from 0.5 to 10 m/s. The positive control was the humeral head, and the negative control was the deltoid muscle belly. A 2-dimensional quality measurement map was used to assess the quality of shear wave propagation for data acquisition and data processing.^12^ When acoustic radiation force impulse is activated, the sampled tissue data are qualitatively and quantitatively evaluated using a proprietary algorithm, and the shear wave velocity is measured in meters per second. High shear wave velocity denotes a “stiff” structure (ie, healthy tendons), whereas low shear wave velocity denotes a less stiff structure (ie, tendinopathic tendons).^8^ SWE tendon stiffness was measured at 1 week, 6 weeks, 3 months, and 6 months postoperatively, but not preoperatively because obtaining preoperative tendon measurements were not possible as the torn tendon edge often retracted beneath the acromion.

### Statistical Analysis

The retear rate at 6 months postoperatively was compared using the Fisher exact test. Kaplan-Meier survival estimates of repair integrity were compared between groups using the log-rank test. Quantitative variables—such as age, tear size area, final follow-up, and patient-rated and surgeon-measured outcomes—were compared between groups using nonparametric Mann-Whitney *U* tests. Categorical variables—such as sex, the proportion of working patients, and retear rate at 6 months—were compared between groups using the χ^2^ or Fisher exact test. Statistical analysis was performed using GraphPad Prism for MacOS Version 8.4.2 (GraphPad Software Inc). Statistical significance was set at *P* < .05. Data are presented as mean ± SEM unless otherwise stated.

## Results

From January 2010 to August 2022, a single surgeon (G.A.C.M.) performed 1478 arthroscopic rotator cuff repairs, of which 244 were revision rotator cuff repairs. In 69 of these repairs, interposition grafts were used to repair partially irreparable tears, and thus they were excluded. In the remaining cohort of 175 revision rotator cuff repairs, 66 patients were insured by workers’ compensation. From this cohort, 19 patients had workers’ compensation approval for and underwent rotator cuff repairs with onlay bioinductive implants, forming the intervention group. In our community, the device was only funded by workers’ compensation insurance.

**Figure 2.**
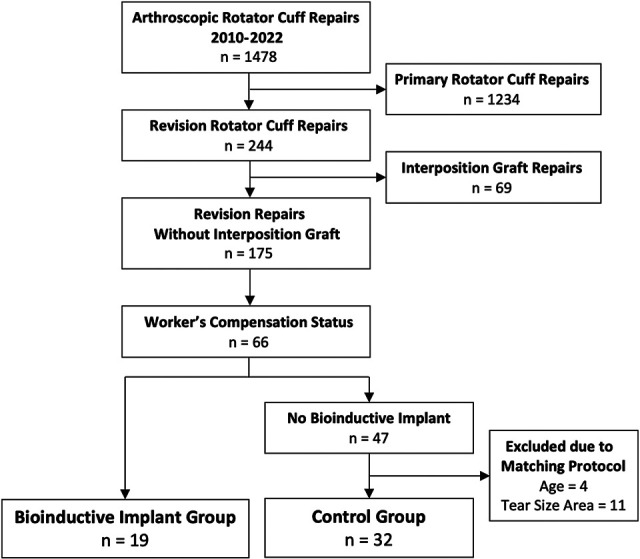
Patient selection flowchart.

The control group was selected from 47 of the remaining patients. In addition to matching for workers’ compensation status, per our matching protocol, groups were matched based on age by excluding 1 patient from the control group because the patient was younger than the youngest patient in the intervention group and by excluding 3 patients in the control group because they were older than the oldest patient in the intervention group. Matching based on tear size area eliminated 3 control group patients with smaller tears than the patient with the smallest tear in the intervention group and 8 patients with larger tears than the patient with the largest tear in the intervention group. Once the above matching process had been completed, there was no statistically significant difference in age or tear size area between the remaining 32 patients in the control group and the 19 patients in the intervention group (Figure 2 and Table 1).

**Table 1:** Patient Characteristics*^a^*

	Bioinductive Implant (n = 19)	Control (n = 32)	*P*
Age at operation, y	56 ± 2 (38-69)	56 ± 1 (39-68)	.965
Male sex, % (n/N)	89 (17/19)	63 (20/32)	.053
Tear size area, mm^2^	205 ± 35 (64-600)	265 ± 28 (70-600)	.122
Final follow-up, mo	14 ± 2 (6-24)	29 ± 5 (24-120)	.087
Ability to work, % (n/N)			
Preoperatively	32 (6/19)	47 (15/32)	.283
At 6 mo	11 (2/19)	28 (9/32)	.176
At final follow-up	21 (4/19)	47 (15/32)	.080
Preoperative strength, N			
Abduction	19 ± 5	32 ± 5	**.028**
Adduction	41 ± 8	58 ± 8	.060
External rotation	32 ± 6	39 ± 4	.078
Internal rotation	42 ± 8	48 ± 5	.270
Lift-off	16 ± 5	23 ± 3	.217
Preoperative passive ROM, deg			
Forward flexion	104 ± 12	125 ± 8	.195
Abduction	101 ± 13	103 ± 8	.929
External rotation	39 ± 6	44 ± 5	.512
Internal rotation	6 ± 1	7 ± 1	.523
Preoperative patient-rated outcomes*^b^*			
Frequency of activity pain (–)	3.3 ± 0.1	3.5 ± 0.2	.133
Frequency of sleep pain (–)	3.3 ± 0.2	3.3 ± 0.2	.763
Frequency of extreme pain (–)	2.2 ± 0.3	2.4 ± 0.3	.489
Level of pain at rest (–)	1.9 ± 0.3	1.7 ± 0.2	.487
Level of overhead pain (–)	3.3 ± 0.3	3 ± 0.2	.231
Level of sleep pain (–)	2.6 ± 0.2	2.2 ± 0.2	.066
Difficulty with behind-the-back movements (–)	2.6 ± 0.4	2.8 ± 0.8	.915
Difficulty with overhead movements (–)	2.9 ± 0.3	3 ± 0.2	.932
Stiffness (–)	1.3 ± 0.3	2.4 ± 0.3	**.020**
Overall satisfaction (+)	0.9 ± 0.3	1.7 ± 0.2	**.032**

*^a^* Data are presented as mean ± SEM unless otherwise stated; data in parentheses are ranges. Bold *P* values indicate statistically significant differences between groups (*P* < .05). ROM, range of motion.

*^b^* (–), lower scores mean better outcomes; (+), higher scores mean better outcomes.

Of note, the control group had significantly less passive abduction ROM, had less patient-rated stiffness, and were less satisfied with their shoulders preoperatively than patients in the intervention group (Table 1).

### Safety

No postoperative infections, hypersensitivity reactions, nerve injuries, or deltoid disruptions were identified in the bioinductive implant or control groups 6 months after surgery.

### Repair Integrity

#### Six-Month Follow-up

The retear rate was 16% (3/19) in the bioinductive implant group and 19% (6/32) in the control group at 6 months after revision rotator cuff repair, with no significant difference between the 2 groups (*P* = .458). A power calculation (α = .05; power = 0.80) determined that a total sample size of 785 patients was needed to find a 10% difference in the retear rate between groups using G*Power Version 3.1.9.6 (Heinrich Heine University).

#### Survival Times

**Figure 3.**
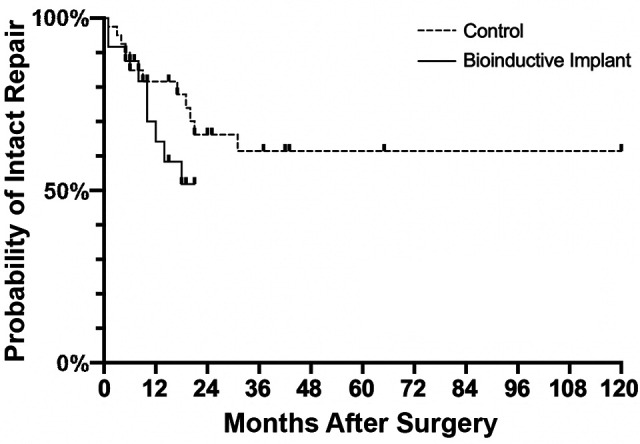
Kaplan-Meier estimates of revision rotator cuff repair survival in the bioinductive implant and control groups.

In the bioinductive implant group, 47% (9/19) of patients were noted to have experienced retears, 2 at 1 month, 1 each at 5 and 8 months, 2 at 10 months, and 1 each at 12, 14, and 18 months postoperatively. In the control group, 38% (12/32) of patients experienced retears, 1 each at 2 months, 3 months, 4 months, and 5 months, 2 at 6 months, and 1 each at 9, 17, 19, 20, 21, and 31 months postoperatively. There was no significant between-group difference in the retear rate at the final follow-up (*P* = .489). In the bioinductive implant group, the median survival time—defined as the time from surgery to the diagnosis of retear on ultrasound assessment—was 14 months compared with 31 months in the control group. There was no significant difference between the 2 groups in the Kaplan-Meier survival estimates (*P* = .289) (Figure 3).

### Shear Wave Elastography (Stiffness)

**Figure 4.**
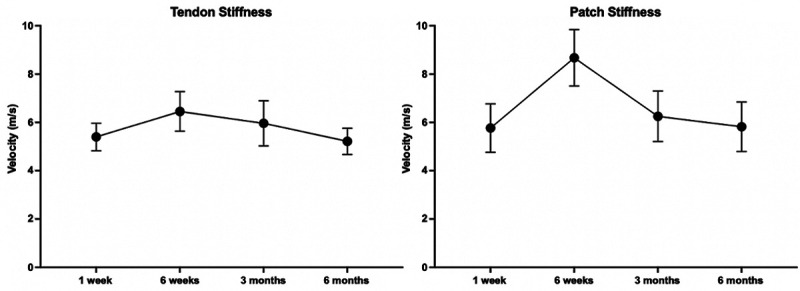
Shear wave elastographic stiffness of the repaired (A) tendon and (B) patch in patients who received bioinductive implants. Data are presented as mean ± SEM.

The SWE stiffness of the repaired tendon remained similar to that of the patch at approximately 6 m/s throughout the first 6 months postoperatively. There was no statistically significant increase in stiffness in either the tendon or the patch in the first 6 months (Figure 4).

### Clinical Outcomes

**Figure 5.**
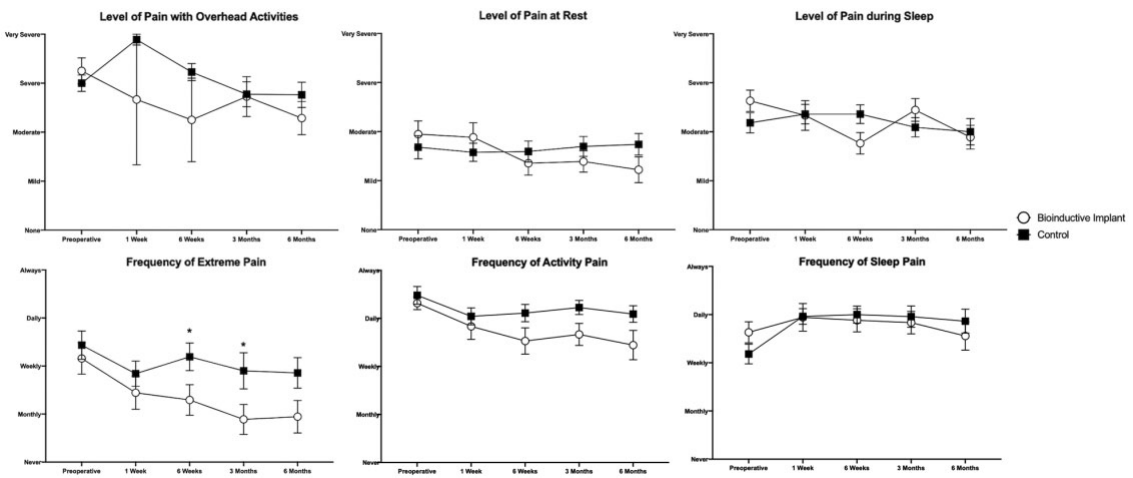
Level and frequency of pain in the bioinductive implant and control groups. Significant differences between groups: *P < .05(Mann-Whitney *U* test). Data are presented as mean ± SEM.

**Figure 6.**
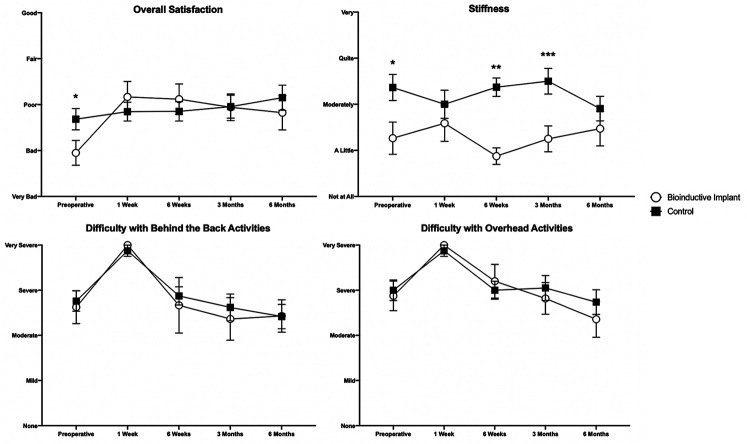
Overall satisfaction and patient-rated stiffness in the bioinductive implant and control groups. Significant differences between groups: *P < .05; **P < .01; ***P < .001 (Mann-Whitney *U* test). Data are presented as mean ± SEM.

**Figure 7.**
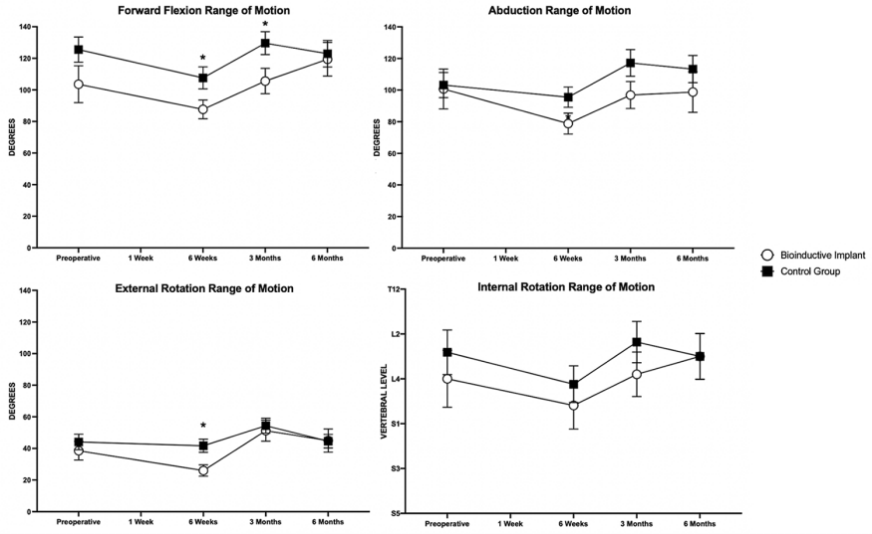
Passive shoulder range of motion in the bioinductive implant and control groups. Significant differences between groups: *P < .05; **P < .01; ***P < .001 (Mann-Whitney *U* test). Data are presented as mean ± SEM.

**Figure 8.**
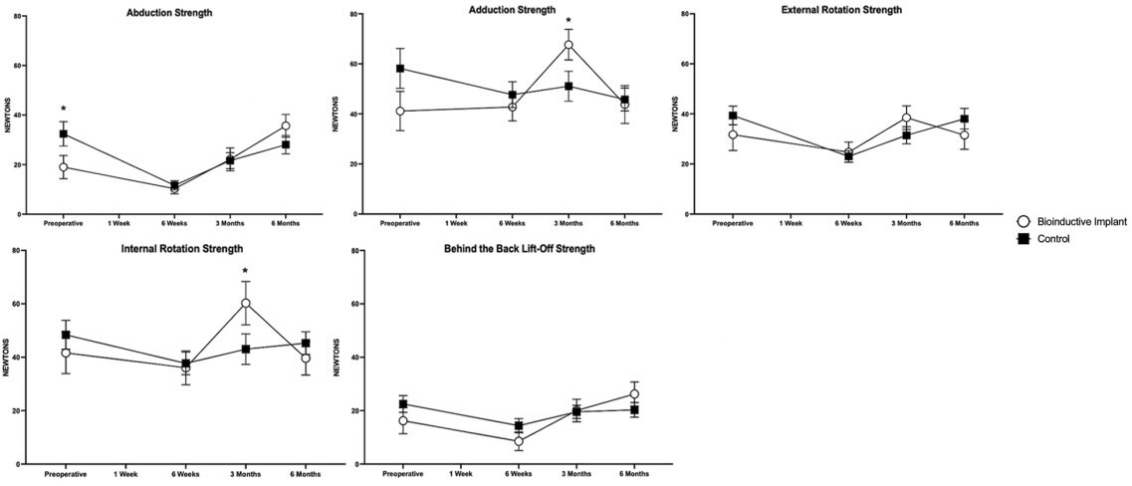
Shoulder strength in the bioinductive implant and control groups. Significant differences between groups: *P < .05 (Mann-Whitney *U* test). Data are presented as mean ± SEM.

At 6 months postoperatively, there were no differences in any patient-rated or surgeon-measured outcomes between the 2 groups (Figures 5-8). Patients in the control group experienced extreme pain more frequently than patients in the bioinductive implant group at 6 weeks (*P* = .034) and 3 months (*P* = .047) postoperatively (Figure 5). Control group patients reported greater shoulder stiffness than patients in the bioinductive implant group preoperatively (*P* = .020) and also at 6 weeks (*P* < .001) and 3 months (*P* = .004) postoperatively (Figure 6).

Patients in the control group had greater passive external rotation ROM at 6 weeks (*P* = .013) postoperatively and greater passive forward flexion ROM at 6 weeks (*P* = .044) and 3 months (*P* = .020) postoperatively than patients in the bioinductive implant group (Figure 7). Patients in the control group were stronger in abduction than patients in the bioinductive implant group preoperatively (*P* = .028), whereas patients in the bioinductive implant group were stronger in internal rotation at 3 months (*P* = .048) and in adduction at 3 months (*P* = .040), respectively. At all other time points, there were no significant differences between groups in either ROM or strength measurements (Figure 8).

## Discussion

We hypothesized that the augmentation of revision rotator cuff repairs with a bioinductive implant would improve the integrity of the repair. However, we found no differences in repair integrity or clinical outcomes between patients who underwent revision arthroscopic repair, regardless of whether the repair was augmented with a bioinductive implant or not.

Case series of rotator cuff repairs augmented with bioinductive implants have shown promising results. Bokor et al^3^ followed 9 patients who underwent arthroscopic rotator cuff repair (8/9 double-row repairs) augmented with a biological collagen implant (Rotation Medical Inc) for full-thickness tears without concurrent capsular release and performed MRI evaluations at 3, 6, 12, and 24 months postoperatively. All repairs in their study remained intact at 24 months, with corresponding improvements in clinical scores from the preoperative to 24-month postoperative periods (*P* < .001).

A subsequent study by Bokor et al^2^ observed 13 patients with intermediate to high-grade partial-thickness rotator cuff tears that were treated using an onlay biological collagen implant (Rotation Medical Inc) after subacromial decompression—without rotator cuff repair. They found MRI evidence of complete healing in 7 of 13 patients at 12 months and progressive improvements in tendon quality in the remaining patients, with no evidence of tear progression in any patients at 24 months.

We found that 47% (9/19) of repairs in the bioinductive implant group and 38% (12/32) of the repairs in the control group had failed, with no difference in Kaplan-Meier survival estimates between groups. In contrast, Thon et al^19^ reported a 96% (22/23) healing rate in patients who underwent rotator cuff repair with a bioinductive implant (Rotation Medical Inc) in their case series. Every patient in the Thon et al series first underwent capsular release, per institutional protocols for repair of large/massive tears or for revision rotator cuff repairs, and received a double-row repair. We used the same bioinductive implants as Thon et al (Rotation Medical Inc was acquired by Smith & Nephew in 2017). However, none of the patients in our study underwent a concurrent capsular release, and all repairs were performed using a single-row inverted-mattress technique. All cases in our study were revisions compared with 7 of the 23 patients in the Thon et al study.

The findings of the present study indicate that over time, a larger sample size might show a statistically significant difference in the retear rate in favor of not receiving a bioinductive implant. The relative success of Thon et al,^19^ compared with our findings, suggests that their excellent results may likely be due to either a better repair technique or because the capsular release induced healing, rather than the addition of a bioinductive implant.

The results of the case series by Thon et al^19^ and Bokor et al^2,3^ that support the use of bioinductive implants were in contrast with the findings of a randomized controlled trial by Iannotti et al^10^ who compared 15 patients who underwent open rotator cuff repair with porcine small intestine mucosa augmentation (Restore Orthobiologic Implant; DePuy) with 15 patients who underwent open rotator cuff repair without biologic augmentation. They found that there was a trend toward a higher retear rate in the bioinductive implant group compared with the control group, as assessed by MRI (4/15 healed in augment vs 9/15 healed without augment; *P* = .11).

Walton et al^23^ similarly showed no difference in repair integrity between patients who underwent rotator cuff repair with versus without bioinductive implants. The intervention group comprised 10 patients who underwent open repair of large/massive rotator cuff tears using porcine small intestine mucosa augment (Restore Orthobiologic Implant), and the control group comprised 12 patients who underwent the same operation without the biological augment. They found that at 2 years postoperatively, 6 of 10 tendons in the biological augment group and 7 of 12 tendons in the control group had retorn per MRI assessment.

The SWE stiffness of tendons has been observed to increase as the tendon heals and restores its material properties.^15^ Another study performed at our institution found that the stiffness of supraspinatus tendons repaired without patches increased by 21% from 1 week to 6 months postoperatively (*P* < .001) and stabilized out to 12 months postoperatively.^9^ However, the SWE stiffness of the tendon in this study remained unchanged at 6 m/s—which is lower than the average of 8 m/s in tendinopathic tendons—and 10 m/s in healthy tendons.^8^

We did not identify any differences in patient-rated or surgeon-measured outcomes between patients with workers’ compensation approval for revision rotator cuff repair who received a bioinductive implant versus those who did not at 6 months postoperatively. Iannotti et al^10^ similarly found no difference in Penn Shoulder Scores between patients who underwent rotator cuff repair with or without porcine small intestine mucosa augmentation.

Furthermore, 4 of 10 patients in the biological augment group in the Walton et al^23^ study experienced severe postoperative reactions that required surgical treatment. Since then, several studies—including this study—have not reported identifying any major complications associated with bioinductive implant augmentation of rotator cuff repairs.^3,5,17,19^

### Strengths and Limitations

A strength of this study was that all patients were consecutively enrolled, and groups were matched for workers’ compensation status, age, and tear size area—with the latter 2 factors being the strongest independent predictors of retear.^11^ The data were prospectively and systematically collected. Furthermore, all patients in this study were operated on by a single surgeon from a single institution that used the same preoperative and postoperative protocols.

A limitation of the present study was that it was only conducted on workers’ compensation patients, which may limit the generalizability of the study, as workers’ compensation status is often a negative prognostic factor. Another limitation was that the study was underpowered to detect a difference in the retear rate between groups, and there was a relatively short mean sonographic follow-up of 14 months in the bioinductive implant group. While longer follow-ups and larger sample sizes may have shown a difference in favor of the bioinductive implant group, Kaplan-Meier analyses of repair integrity indicated a trend favoring the control group. Furthermore, the secondary outcomes were only collected up to 6 months postoperatively, and strength and ROM did not seem to have plateaued by then. Finally, the patients in the 2 sequential cohorts were not prospectively randomized or blinded.

## Conclusion

The addition of an onlay bioinductive implant did not improve the integrity of the repair or the SWE stiffness of the tendon after revision arthroscopic rotator cuff repair in consecutive patients with workers’ compensation claims. Furthermore, there were no differences in patient-rated or surgeon-measured outcomes between the bioinductive implant group and the control group 6 months after surgery.

## Figures and Tables

**Figure A1.**
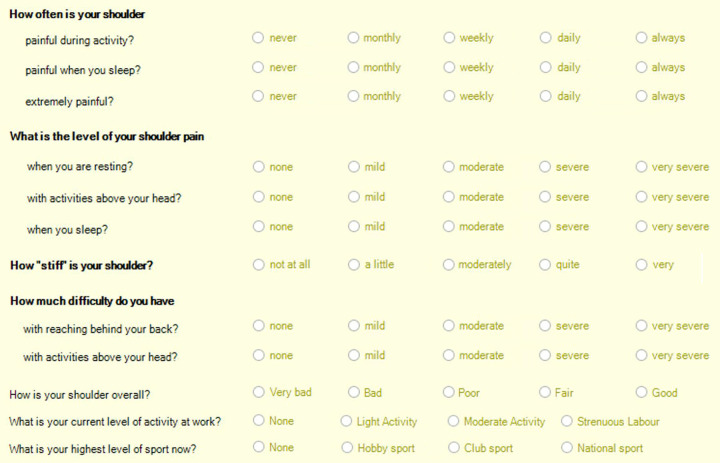
Twelve-item patient-rated outcome questionnaire.
